# Elimination Kinetics of Ethanol in a 5-Week-Old Infant and a Literature Review of Infant Ethanol Pharmacokinetics

**DOI:** 10.1155/2013/250716

**Published:** 2013-12-04

**Authors:** Jonathan B. Ford, Mac T. Wayment, Timothy E. Albertson, Kelly P. Owen, Joshua B. Radke, Mark E. Sutter

**Affiliations:** ^1^Department of Emergency Medicine, School of Medicine, University of California, Davis, 4150 V Street, PSSB 2100, Sacramento, CA 95817, USA; ^2^Department of Pediatrics, School of Medicine, University of California, Davis, 2516 Stockton Boulevard, Suite 221, Sacramento, CA 95817, USA; ^3^Department of Internal Medicine, School of Medicine, University of California, Davis, 4150 V Street, PSSB 2100, Sacramento, CA 95817, USA

## Abstract

Primary ethanol metabolism occurs through alcohol dehydrogenase, but minor metabolic pathways such as the P450 enzymes CYP2E1 and CYP1A2 and the enzyme catalase exist. These enzymes have distinct developmental stages. Elimination kinetics of ethanol in the infant is limited. We report the elimination kinetics of ethanol in a 5-week-old African-American male who had a serum ethanol level of 270 mg/dL on admission. A previously healthy 5-week-old African-American male was brought to the ED with a decreased level of consciousness. His initial blood ethanol level was 270 mg/dL. Serial blood ethanol levels were obtained. The elimination rate of ethanol was calculated to be in a range from 17.1 to 21.2 mg/dL/hr and appeared to follow zero-order elimination kinetics with a *R* ^2^ = 0.9787. Elimination kinetics for ethanol in the young infant has been reported in only four previously published reports. After reviewing these reports, there appears to be variability in the elimination rates of ethanol in infants. Very young infants may not eliminate ethanol as quickly as previously described. Given that there are different stages of enzyme development in children, caution should be used when generalizing the elimination kinetics in young infants and children.

## 1. Introduction

Alcohol intoxication in children and infants (less than 12 months of age) is uncommon compared to teenagers and adults. These children are often observed in the intensive care unit (ICU) to monitor for adverse events such as hypoglycemia, seizures, and failure to protect their airway [1]. Currently, there is little data to help clinicians predict their clinical course.

Ethanol is metabolized primarily by alcohol dehydrogenase (ADH) but minor metabolic pathways such the cytochrome P450 enzymes CYP2E1 and CYP1A2 and the enzyme catalase exist [2]. These enzymes have distinct developmental stages. The ethanol elimination rate of an average-sized nontolerant adult is 15–20 mg/dL/hr [2]. Previous reports suggest that children aged 7 months to 10 years old have more rapid elimination rates [1, 3, 4]. However, little is known about the elimination rates in very young infants. We report the elimination kinetics of ethanol in a 5-week-old male who had a serum ethanol level of 270 mg/dL on admission.

## 2. Case Report

A 5-week-old African-American male infant was brought to the emergency department (ED) with a depressed level of consciousness. He was in his usual state of health until approximately 30 minutes prior to admission when the child's mother found him lethargic. She thought that he smelled like alcohol. According to her, a family member had been drinking vodka while caring for the patient. The patient's medical history was pertinent for a healthy full-term gestation with normal postnatal weight gain. Upon admission to the emergency department, his vital signs were within normal limits except for a temperature of 36.1 degrees Celsius. He weighed 4.3 kilograms. On exam, he was obtunded but was ventilating adequately. His breath smelled of alcohol. A point-of-care (POC) fingerstick blood glucose was 63 mg/dL. His serum ethanol level obtained approximately 1 hour after the presumed time of ingestion was 270 mg/dL. He was admitted to the pediatric intensive care unit (PICU) for observation and treatment with glucose containing intravenous fluids. The infant recovered uneventfully without requiring ventilation assistance and was back to normal about 24 hours later. Serial ethanol levels were obtained and shown below in Table 1.

**Figure 1:**
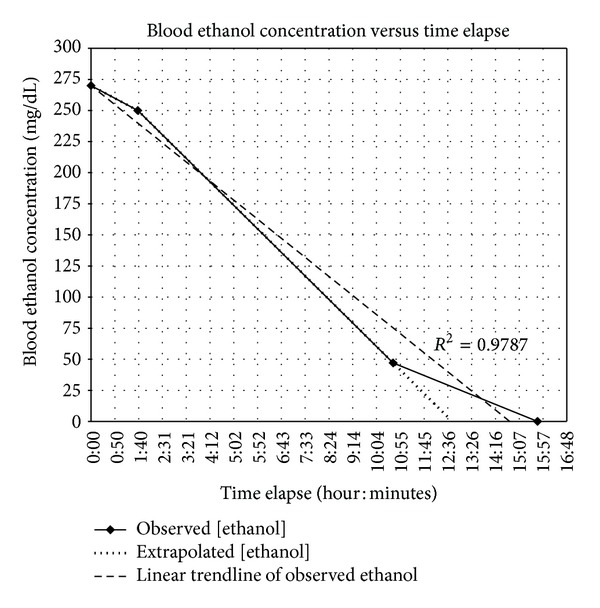
Blood ethanol concentration (mg/dL) versus time (hr : min). (—) represents actual measure concentrations of ethanol and their corresponding times. (⋯) represents an extrapolated line measured from the previous 2 points to show that if the infant had continued linear elimination, he would have completely eliminated the ethanol at 12 hours and 45 minutes. (- - -) represents a linear trendline applied to the actual measured ethanol concentrations and their corresponding times to suggest linear, or zero-order, elimination kinetics with a coefficient of determination (*R* ^2^) = 0.9787.

We were able to obtain 4 serum blood levels for our patient, 3 of which had detectable ethanol (Figure 1). It is estimated that the ethanol exposure was approximately 1 hour prior to the first blood draw to compensate for the time when he was last seen normal, ambulance transport, ED triage, and venipuncture. Thus, his peak blood ethanol level may have been higher. The observed time at which ethanol was no longer detected may not represent the actual time at which ethanol elimination was complete in the patient. Therefore, we extrapolated the slope of the line between the 2nd and 3rd blood level to show that if the patient continued at a steady rate, he could have achieved complete elimination at about 12 hours and 45 minutes after admission. Using both the recorded time and the extrapolated time to complete elimination, it is estimated that our infant had an ethanol elimination rate in the range of 17.1 mg/dL/hr to 21.2 mg/dL/hr, respectively. A linear trendline applied to the recorded data revealed a correlation coefficient (*R* ^2^) of 0.9787 indicating very good fit. Finally, using the updated Widmark equation [5], an assumed volume of distribution (Vd) of ethanol in a term infant of 0.75, L/kg [3], the initial blood ethanol level obtained, and the alcohol content in vodka of 40% alcohol by volume, it is estimated that our infant was exposed to more than 29 mL or 1 oz of vodka.

## 3. Discussion

Infants and children may have varying rates of ethanol elimination due to different stages in development of metabolic enzymes. A study using fetal liver tissue obtained from legal abortions determined that ADH is present in the human fetal liver by the end of the first trimester [6]. However, this study found that the amount of ADH and its activity is significantly lower in infants than in adults [6]. Adult ADH levels and activity are present near the age of 5 years old [6]. The activity of CYP2E1 has been reported to be only 30–40% of adult activity until 1 year of age while the activity of CYP1A2 is about 5% of adults in the neonatal period and 25% of adults by 1 year of age [7]. In another study of human liver tissue, the concentration of catalase was equal to or significantly higher in neonates (10 days to 32 weeks) compared to adults [8]. The authors of this study concluded that catalase might play a more predominant role in the metabolism of alcohols in the perinatal infant. Finally, genetic polymorphisms can influence the development of all the above enzymes, particularly ADH, and lead to individual variability in ethanol metabolism [9].

In review of the literature, many reports on ethanol intoxication in children age 7 months to 10 years suggest that children eliminate ethanol more rapidly than adults [1, 3, 4]. However, actual pharmacokinetics of ethanol in young infants are reported in only four previous publications. A 3-month-old infant with an initial blood ethanol level of 338 mg/dL was found to have an ethanol elimination rate of 21 mg/dL/hour [10]. A 7-month-old infant with an initial blood ethanol level of 183 mg/dL was found to have an ethanol elimination rate of 49.7 mg/dL/hr [3]. A recently published case series of two infants, both 8 weeks of age, described a detailed account of their ethanol intoxication [11]. Both patients presented with an acute life-threatening event (ALTE). The first patient was found to have a blood ethanol level of 278 mg/dL and metabolized the ethanol in about 12 hours. The other patient had a blood ethanol level of 405 mg/dL and metabolized the ethanol in about 21 hours [11]. After dividing the patient's initial blood ethanol concentration by the approximate number of hours it took to metabolize the ethanol, these patients had an overall elimination rate of approximately 23 and 19 mg/dL/hr, respectively. With the exception of the 7-month-old infant, the other published infants, including ours, had similar overall elimination rates. This rate is slower than previous reports of older children and may reflect decreased ADH levels. However, the rate is similar to that of a normal-sized nontolerant adult. This may be due an increased dependence on the catalase pathway.

Alcohol follows zero-order elimination kinetics, or a fixed amount per unit time independent of concentration, in adults [2]. Previously published reports on infant kinetics suggest that infants may follow first-order elimination kinetics or a percent of concentration per unit time [3, 10, 12]. McCormick et al. demonstrated a transition from 1st-order to zero-order kinetics at a blood ethanol level of approximately 225 mg/dL in the two infants they observed [11]. Our patient appeared to follow a constant elimination rate consistent with zero-order kinetics given the linear trendline with a coefficient of determination (*R* ^2^) value of 0.9787. However, we acknowledge that determining first-order or zero-order kinetics from our data is limited by the few number of blood ethanol levels obtained.

## 4. Conclusion

Our case represents the youngest reported infant ethanol intoxication with pharmacokinetic data. The 5-week-old infant we observed had an initial blood ethanol level of 270 mg/dL and had an estimated ethanol elimination rate in a range from 17.1 to 21.1 mg/dL/hr that seemed to follow zero-order kinetics. He had no adverse events such as hypoglycemia, seizures, or apnea. This case provides additional insight into the elimination of ethanol by young infants and suggests that very young infants may not eliminate ethanol as quickly as older infants and children. There is likely a spectrum of elimination rates of ethanol between the newborn period to older infants based on enzyme expression and the role of catalase, with young infants eliminating ethanol less efficiently than older infants. Given the large variability of elimination rates in the very few published case reports, caution should be used when generalizing about the elimination kinetics in young infants and children.

## Figures and Tables

**Table 1:** Blood ethanol concentration of 5-week-old male showing time of laboratory analysis.

Time	Blood ethanol concentration (mg/dL)
11:34 PM	270
01:13 AM	250
11:53 AM	47
03:20 PM	0
